# Responses of soil bacterial composition and network complexity to O_3_ stress and straw return in a soybean cropland

**DOI:** 10.3389/fpls.2026.1846591

**Published:** 2026-08-12

**Authors:** Qun Gang, Yan Wang, Jing-Shi Li, Bing Mao, Tian-Hong Zhao

**Affiliations:** 1 College of Agronomy, Shenyang Agricultural University, Shenyang, China; 2 CAS Key Laboratory of Forest Ecology and Management/Daqinggou Ecological Station, Institute of Applied Ecology, Chinese Academy of Sciences, Shenyang, China; 3 Key Laboratory of Environment Change and Resources Use in Beibu Gulf, Ministry of Education, Nanning Normal University, Nanning, China

**Keywords:** bacterial community composition, bacterial diversity, O3 stress, Soybean, Straw return

## Abstract

Soil microbial community and function play a key role in root nutrient acquisition and growth. Yet, the combined effects of O_3_ stress and straw return on soil microbial composition and network complexity remain unclear. Here, a pot experiment was conducted in open-top chambers to monitor the responses of soil microbial diversity, composition, and network complexity at branching and podding stages of soybean (*Glycine* max; a species highly sensitive to O_3_ stress) to O_3_ stress (45 ± 5 ppb; 80 ± 10 ppb; and 110 ± 10 ppb) and straw treatment (no straw return and straw return). Under O_3_ stress at 110 ± 10 ppb, SOC and microbial biomass C and N significantly increased at the branching stage and decreased at the podding stage. Under O_3_ stress, the Chao1 and PD indices, along with the number of observed species, were significantly increased by straw return. The bacterial network complexity was negatively affected by O_3_ stress (80 ± 10 ppb) and positively affected by straw return. Microbial diversity (Chao1, Shannon, and PD indices) showed greater total standard effects than the factors of soil stoichiometry (C/N, C/P, and N/P ratios). Straw return had the lowest total standard effect on microbial network community and complexity than the other factors. Collectively, the slight alleviating effect of straw return on the reduced microbial topology induced by O_3_ stress was mainly associated with bacterial functions rather than soil physicochemical properties.

## 1 Introduction

Soybean (*Glycine max* L. Merr), which is one of the crops most sensitive to elevated O_3_ among food crops (Burkey and Carter, 2009; Mills et al., 2006), is the major legume crop in Northeast China. The planting area of soybean accounts for 33% of the nation’s total planting area (Liu and Herbert, 2002). Due to the rapid development of industrialization and population expansion, O_3_ concentrations exceeded the WHO (World Health Organization) standard of 100 μg m^−3^ on more than 30% of the days in 2013–2015 in China (Gong et al., 2018; Wang et al., 2017). These recorded O_3_ levels are beyond the acceptable standards for safe concentrations of O_3_ in air and are known to be detrimental to leaf photosynthesis and soybean crop yield (Shang et al., 2017; Wittig et al., 2009; Pregitzer et al., 2008).

The soil microbial community plays a crucial role in driving various processes, is recognized as a key factor in improving soil properties, and enhances plant growth and yield (Mendes et al., 2017; Geisen et al., 2022). Soil microbial diversity, community, and function are known to be sensitive to environmental changes (Pescador et al., 2022). For instance, elevated O_3_ has adverse effects on soil microbial biomass and the α-diversity of bacterial, archaeal, and fungal groups (Bao et al., 2015; Chen et al., 2019; Wang et al., 2019). Elevated O_3_ could reduce the allocation of carbon (C) derived from the soil, thereby reducing the availability of nutrients for soil microbes and negatively affecting belowground processes driven by microbes (Agathokleous et al., 2020). In contrast, some studies have found that elevated O_3_ could stimulate the bacterial α-diversity in agro-ecosystems (Feng et al., 2015; Li et al., 2015; Zhang et al., 2019). Yet, the contrasting results are mainly due to the different crop species and environment (Innes et al., 2004; Tomazelli et al., 2023).

Direct straw returning is a primary tillage practice for crop cultivation. After returning, straw can improve soil structure, reduce soil bulk density, activate soil nitrogen and organic phosphorus, and improve yields (e.g., Tan et al., 2007; Zhao et al., 2015). Despite its beneficial effects, the mechanism underlying the stimulation of belowground biological pathways by straw return that improve soil quality remains ambiguous, which might be due to the complex composition and activities of the soil microbial community (Fierer, 2017). In straw-return soils, microbial biomass, richness, and diversity of the microbial community are substantially elevated, and their composition is altered (Chen et al., 2017; Li et al., 2021; Liu et al., 2023). For instance, the ANOVA and non-metric multidimensional scaling (NMDS) results of Li et al. (2021) suggested that straw retention was the main factor regulating bacterial alpha diversity and reshaping the bacterial community composition in maize (Zea mays L.). In contrast, Liu et al. (2023) found that straw return significantly affected the microbial beta-diversity but not microbial alpha-diversity in a rice-wheat rotation system. Currently, soybean is increasingly affected by elevated O_3_ stress (Mao et al., 2017, 2018, 2022). Considering the crucial role of the soil microbial community in driving various processes (Meena et al., 2015; Kumar et al., 2016), it is necessary to evaluate the responses of the soil microbial community and function to the interaction between O_3_ stress and straw return in order to assess the impacts of elevated O_3_ on crop yield and nutrient utilization in cropland ecosystems under actual tillage practice conditions. However, the microbial mechanism of the interaction effect of O_3_ stress and straw return on soybean growth remains largely unclear.

Many studies on soil microbial processes have mainly focused on bacterial diversity and community composition. Interactions among microbial communities are also essential for ecosystem functioning, and they provide important insights into community structure (Wagg et al., 2019). However, the interactions among soil bacterial communities under environmental change have been largely ignored. Co-occurrence ecological network analysis can identify potential interactions among microbial taxa, shared physiologies, or habitat affinities (Dini-Andreote et al., 2014). Therefore, network analysis is increasingly recommended to investigate specific microbial interactions within complex environments (Jansson and Hofmockel, 2020; Shannon et al., 2003; Wang et al., 2022). Studying microbe–microbe interactions allows a better understanding of their functioning, vulnerability to environmental changes, and adaptive strategies. Recently, few studies have used network analysis to evaluate the influence of elevated O_3_ and straw return on soil microbial co-occurrence networks. For instance, Zhang et al. (2019) found that elevated O_3_ induced changes in the soil bacterial co-occurrence network. Wang et al. (2023) found that elevated O_3_ decreased the network complexity of the soil bacterial community. Meanwhile, Xu et al. (2023) found that the co-occurrence network in straw-amended soil exhibited greater complexity. However, how the co-occurrence network of bacterial communities responds to the interaction influence of elevated O_3_ and straw return remains elusive.

In addition, previous studies have shown that soybean yield is mainly determined by the growth phase, such as the branching and podding stages (Egli, 2010). The subsequent changes in soil microbial community and function caused by O_3_ stress and/or straw return might differ considerably among different stages of soybean. Knowledge is still scarce regarding the responses of the soil microbial network community and network complexity at different developmental stages of soybean to O_3_ stress and/or straw return. Therefore, we hypothesized that (1) elevated O_3_ stress would significantly and negatively affect the bacterial network community and complexity; (2) straw return could mitigate the negative effects of O_3_ stress; and (3) the mitigation effect of straw return on the network community and network complexity would be mediated by changes in soil MBN and microbial diversity. To test these hypotheses, the branching and podding stages of soybean were chosen as sampling times to evaluate the soil microbial diversity and composition in response to O_3_ stress and/or straw return using a pot experiment in open-top chambers (OTCs). Meanwhile, the soil bacteria microorganisms at each growth stage were holistically analyzed using group-specific high-throughput sequencing approaches. The distinct patterns within the microbiomes in the soil with and without straw under three levels of O_3_ stress (45 ± 10 ppb, 80 ± 10 ppb, and 110 ± 10 ppb) were determined. The microbial associations were explored through co-occurrence network analyses to determine the effects of O_3_ stress and straw incorporation on the ecological interactions. The mechanisms by which O_3_ stress and straw incorporation affect the ecological processes governing soil microbial community assembly were further investigated. Our findings will help improve understanding of the association between soil microbiomes and the interactions of O_3_ stress and straw incorporation in soybean croplands.

## 2 Materials and methods

### 2.1 Experimental site

The experimental site was located at the National Field Observation and Research Station of Agro-ecosystems in Shenyang, Liaoning Province, China (41°31′N, 123°24′E). This region has a continental monsoon climate with a mean annual temperature of 7.0°C–8.0°C, annual precipitation of 650–700 mm, and an annual non-frost period of 147–164 days. The soil at the study site is classified as an Alfisol (US Soil Taxonomy) with 11.28 g kg^-1^ organic C, 1.20 g kg^-1^ total nitrogen (N), 0.41 g kg^-1^ total phosphorus (P), and a pH (H_2_O) of 6.7 at a depth of 0–15 cm.

### 2.2 Experimental design and sampling

The study was conducted on soybean plants grown in OTCs from 16 June to 30 August, 2017. The OTCs, which were established in 2008, had a mean daytime temperature of approximately 25.6°C ± 3.7 °C, a mean daytime relative air humidity of 50.6% ± 19.9%, a mean O_3_ concentration of approximately 45 ± 5 ppb, and an average AOT40 (the O_3_ concentration accumulated over a threshold O_3_ concentration of 40 ppb during daylight hours) of 3.5 ppm h^-1^ during clear-sky conditions from June to August (Mao et al., 2022). O_3_ was produced from pure oxygen with an O_3_ generator (GP-5J Guolin Ltd., Qingdao, China). The O_3_ concentrations were continuously monitored by O_3_ analyzers (S-900 Aeroqual Ltd., Auckland, New Zealand) every day during the whole day from 16 June to 30 August, 2017, and were controlled by computers using a professional program for O_3_ dispensing and monitoring (Mao et al., 2022).

The experiment was conducted using three O_3_ treatments (ambient: 45 ppb, 80 ppb, and 110 ppb) combined with two straw treatments (straw return and no straw return). The experimental design was based on completely randomized plots and the following six treatments (three O_3_ treatments × two straw treatments) at the branching stage (BI) and podding stage (BF), respectively: 1) T0S0: O_3_ concentration 45 ± 10 ppb (T0) and no straw return (S0); 2) T0S1: O_3_ concentration 45 ± 10 ppb (T0) and straw return (S1); 3) T1S0: O_3_ concentration 80 ± 10 ppb (T1) and no straw return (S0); 4) T1S1: O_3_ concentration 80 ± 10 ppb (T1) and straw return (S1); 5) T2S0: O_3_ concentration 110 ± 10 ppb (T2) and no straw return (S0); and 6) T2S1: O_3_ concentration 110 ± 10 ppb (T2) and straw return (S1). There were a total of nine OTCs (three O_3_ treatment × three replicates), with 12 pots in each OTC (two collected periods, namely, branching stage and podding stage) × two straw treatments × three replicates).

The potted soybean cultivar was Tiefeng 29, which was seeded in each pot (26 cm long × 36 cm wide × 45 cm deep) on 9 May 2017. According to the average soybean yield and potted area, soybean straw (20 g oven-dried leaves and stems), which was subjected to O_3_ fumigation in 2016, was crushed and applied *in situ* to a depth of 20 cm per pot. The soil used in each pot experiment was collected from a cropland (at the 0–15 cm layer) at the study site before crops were planted. After sieving (<2 mm), the soil was immediately used to prepare the pot experiment. The previous crop was soybean (Tiefeng 29), and the field did not receive any N fertilization or P fertilization because of fallow management before the beginning of the experiment. NH_4_H_2_PO_4_ was applied at an application rate of 300 kg ha^-1^ before sowing in each plot. The plants were irrigated daily to avoid water stress. Appropriate measures were taken to keep the plants free from any biotic stresses, diseases, and weed. Five plants were planted in each pot (26 cm long × 36 cm wide × 45 cm deep). The soybean used in our study was neither herbicide-tolerant nor genetically modified.

Soil samples (rhizosphere soil; 20cm) were collected at the branching stage (10 July 2017) and podding stage (30 August 2017). Five soil samples from each pot were randomly collected by using a soil corer with an inner diameter of 4.5 cm and were pooled together to form one composite sample. The collected soil samples were immediately sieved (< 2 mm) to remove visible stones, roots, and plant materials, and were then divided into three subsamples. The first subsample was air-dried at 25°C for the analysis of soil physicochemical properties, the second subsample was stored at 4°C for enzyme analysis, and the third subsample was freeze-dried and stored at –20°C for microbial community analysis.

### 2.3 Soil physicochemical and biochemical properties analysis

Soil organic carbon (SOC) was analyzed using the K_2_Cr_2_O_7_-H_2_SO_4_ calefaction and titration method (Nelson and Sommers, 1982). Dissolved organic nitrogen (DON) and total P concentration were determined using an elemental analyzer (Vario MAX CNS, Elementar Analysensysteme GmbH, Hanau, Germany) (Zhao et al., 2017). Microbial biomass carbon (MBC), microbial biomass nitrogen (MBN), and microbial biomass phosphorus (MBP) of soybean soil were analyzed using the chloroform fumigation–extraction method described by Vance et al. (1987) and Brookes et al. (1985). Microbial C flush, N flush, and P flush were converted to microbial biomass C (MBC), N (MBN), and P (MBP) using conversion factors of 0.45, 0.54, and 0.40, respectively.

To measure enzyme activities (invertase activity, polyphenol oxidase activity, peroxidase activity, and acid phosphomonoesterase activity), the UV–visible spectrophotometry method was used in the present study (Frankeberger and Johanson, 1983; Perucci et al., 2000). Briefly, the invertase activity was determined through 3, 5-dinitrosalicylic acid colorimetry and expressed as mg of glucose in 1 g of soil C within 24 h. Polyphenol oxidase activity was determined using the pyrogallol colorimetric method, and its activity was expressed as mg gallic acid in 100 g soil C after 3 h. Peroxidase activity was determined by the purple concentration of 1 g soil C after 2 h. Acid phosphomonoesterase was determined from the p-nitrophenol concentration after incubation with p-nitrophenyl phosphate (PNP) for 1 h at 37°C (Tabatabai and Bremner, 1969).

### 2.4 Detection of soil bacterial community composition

The bacterial DNA was extracted from 0.5 g of soil samples using E.Z.N.A. soil DNA kits (OMEGA, USA) according to the manufacturer’s instructions. The V3–V4 region of the 16S rRNA gene was amplified with the primers 338F (5′ -ACTCCTACGGGAGGCAGCAG-3′) and 806R (5′-GGACTACNNGGGTATCTAAT-3′) (Fadrosh et al., 2014). After purifying PCR amplification products, PCR was conducted in 10 ng of DNA, 0.2 mg/mL of BSA, 0.2 μmol of each primer, and 12.5 μL of SYBER Green Master Mix. The amplicons were extracted from the 2% agarose gel and purified using the AxyPrep DNA Gel Extraction Kit (Axygen Biosciences, Union City, CA, USA). They were then quantified using a Quantifluor™-ST Fluorometer (Promega, USA).

Sequencing was performed using the Illumina MiSeq 300 PE platform (Allwegene Company, Beijing, China). The raw sequencing data were filtered and pruned using PReprocessing and INformation of SEQuence data (PRINSEQ) and Mothur tools. The UCLUST tool in Quantitative Insights Into Microbial Ecology (QIIME) was used to conduct a cluster analysis on sequences with a sequence similarity threshold of 97%, and operational taxonomic units (OTUs) with biological significance were classified based on the species annotation in the Greengenes 16S rRNA database. Before diversity analysis, the ASV abundance tables were rarefied. The Chao1 richness index, Shannon diversity index, PD index, and observed species were calculated using QIIME (V 1.9.1). Raw MiSeq high-throughput sequencing data were submitted to the SRA database on the NCBI website, with the search number PRJNA1053905 (https://www.ncbi.nlm.nih.gov/sra/PRJNA1053905).

### 2.5 Statistical analysis

One-way analysis of variance (ANOVA) was performed to compare differences in soil physicochemical and biochemical properties and microbial diversity across the six O_3_ stress and straw return treatments at the branching and podding stages. Tukey’s multiple range tests were applied for pairwise comparison of the means (± SE). Then, a multivariate analysis of variance (general linear model (GLM)) was used to evaluate the single effects of growth stage, O_3_ stress, and straw return, along with their possible interactions, on soil physicochemical and biochemical properties and microbial diversity (SPSS 16.0). In this study, we assessed three soil physicochemical and biochemical functions: soil physicochemical properties (SOC, DON, P, and pH), soil microbial biomass (microbial biomass C, N, and P), and soil enzyme activity (invertase, polyphenol oxidase, peroxidase, and acid phosphomonoesterase). Prior to statistical analysis, the data were subjected to natural log transformation. To quantify ecosystem multifunctionality, we calculated the average index according to previous studies (Delgado-Baquerizo et al., 2020; Xiao et al., 2024).

Random Forest analysis was performed using the “randomForest” package (R 4.2.1) to regress soil physicochemical and biochemical properties on soil microbial alpha diversity. The percentage increase in the mean squared error (%IncMSE) was used to evaluate the relative importance of independent variables in predicting bacterial diversity. Non-metric multidimensional scaling (NMDS) based on Bray–Curtis dissimilarities was applied to visualize variations in bacterial community composition across O_3_ stress and straw return treatments.

The underlying co-occurrences between bacterial taxa via network analysis (MENA; http://ieg4.rccc.ou.edu/MENA) based on random matrix theory were performed to evaluate the effects of O_3_ stress and straw return on the soil microbial community in soybean (Deng et al., 2012). The OTUs retrieved from 16s rRNA gene sequencing were used for MENA. For each slope direction and position, networks were built from the OTUs (thresholded at relative abundance >0.01%) using Pearson correlation (|r| > 0.6, *P* < 0.01). A similar cutoff value (0.80–0.88) was used to construct the network. Various network topological features, including nodes, degree, links, average path distance, geodesic efficiency, density, and clustering coefficient, were calculated (Zhou et al., 2020). The network was visualized using Gephi (version 0.9.2) (Bastian et al., 2009). Topological characteristics were used to describe the complexity of the co-occurrence networks, including the edge number (connection number of all nodes), average degree (average number of edges per node), average clustering coefficient (the extent of the module structure present in a network), average path distance (average value of the distances between every two nodes), and network diameter (shortest distance between the two most distant nodes) (Fan et al., 2020; Gao et al., 2022). Pearson’s correlation analyses between soil microbial community composition (the module1–3 calculated by co-occurrence networks) and soil physicochemical and biochemical properties were performed (origin 2021). To identify potential core species in the network, we calculated the within-module connectivity (Zi) and among-module connectivity (Pi) of each node. According to established criteria, we identified module hubs (Zi ≥ 2.5, Pi < 0.62), connectors (Zi < 2.5, Pi ≥ 0.62), and network hubs (Zi ≥ 2.5, Pi ≥ 0.62), which are referred to as keystone nodes (Deng et al., 2012).

A structural equation model (SEM) was used to evaluate the relationships among soil physicochemical and biochemical properties, soil enzyme activity, soil microbial diversity, and soil microbial community composition under O_3_ stress and straw return treatments. Soil enzyme activity variables were created by polyphenol oxidase activity (PPO) and peroxidase activity (POX). Chao1 index, Shannon index, and PD index were used to determine the microbial diversity, and the module1–3 calculated by co-occurrence networks was used to determine the microbial community composition. These variables were inserted into AMOS 17.0 (SPSS, Chicago, IL, USA) for SEM construction and analysis. Chi-square (χ^2^; good fit: 0≤ χ^2^ ≤2), *P*-value (0.05 ≤ *P* ≤ 1.00), goodness-of-fit index (GFI > 0.90), and the root mean square error of approximation (RMSEA < 0.05) were measured (Grace and Keeley, 2006).

## 3 Results

### 3.1 Soil physicochemical and biochemical properties

**Figure 1:**
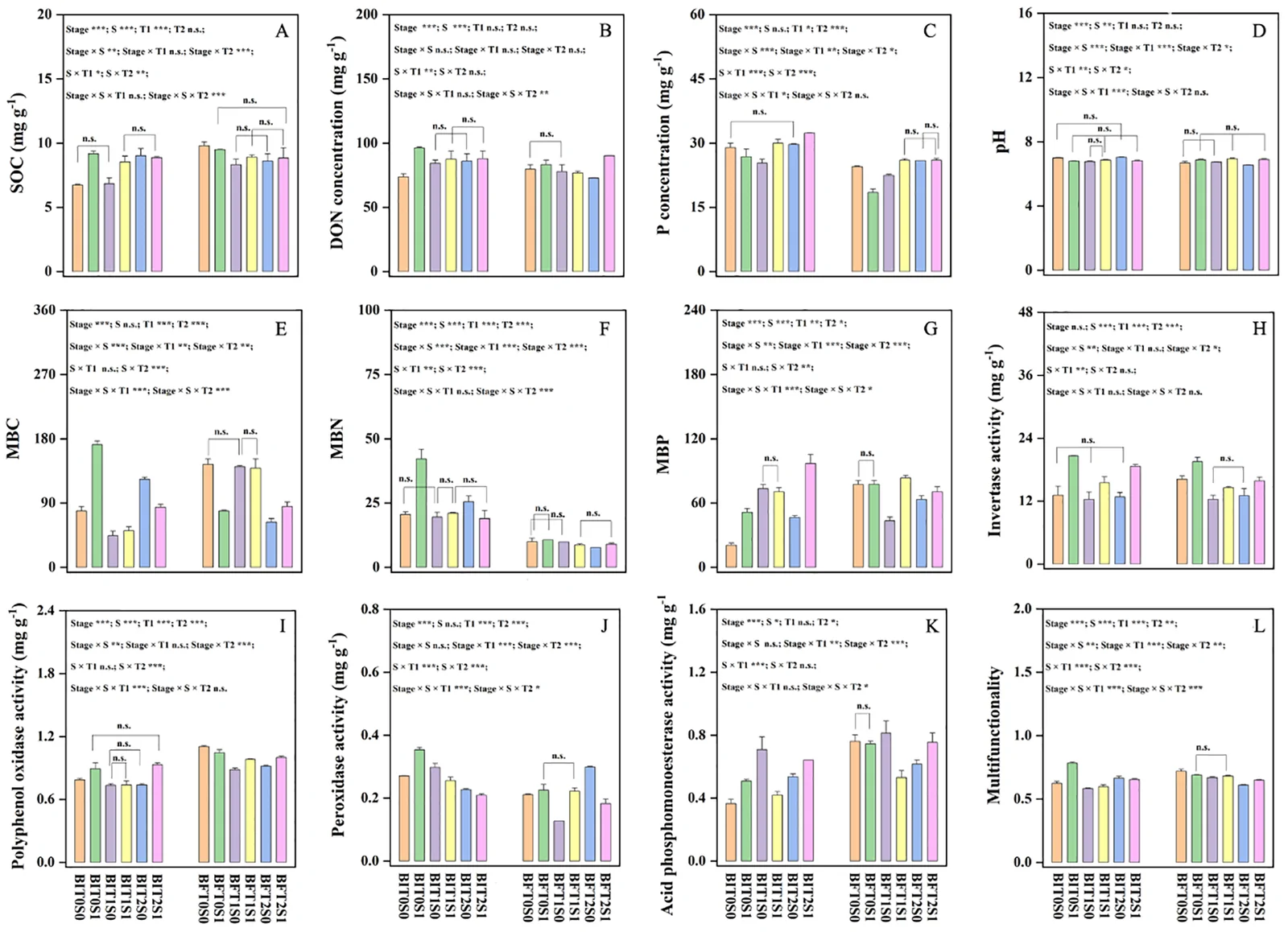
Soil physicochemical properties **(A–D)**, soil microbial biomass **(E–G)**, soil enzyme activities **(H–K)**, and multifunctionality of soil physicochemical properties and soil microbial biomass **(L)** under O_3_ stress and straw return at the branching and podding stages. Data are shown as mean ± standard errors (n = 3). For each parameter, results of the general linear model (GLM) are reported, with asterisks showing the significant effect of O_3_ stress (T1: O_3_ concentration 80 ± 10 ppb; T2: O_3_ concentration 110 ± 10 ppb), straw return (S), growth stage (stage), and their interactions. BIT0S0: O_3_ concentration 45 ± 10 ppb and no straw return at the branching stage; BIT0S1: O_3_ concentration 45 ± 10 ppb and straw return at the branching stage; BIT1S0: O_3_ concentration 80 ± 10 ppb and no straw return at the branching stage; BIT1S1: O_3_ concentration 80 ± 10 ppb and straw return at the branching stage; BIT2S0: O_3_ concentration 110 ± 10 ppb and no straw return at the branching stage; BIT2S1: O_3_ concentration 110 ± 10 ppb and straw return at the branching stage; BFT0S0: O_3_ concentration 45 ± 10 ppb and no straw return at the podding stage; BFT0S1: O_3_ concentration 45 ± 10 ppb and straw return at the podding stage; BFT1S0: O_3_ concentration 80 ± 10 ppb and no straw return at the podding stage; BFT1S1: O_3_ concentration 80 ± 10 ppb and straw return at the podding stage; BFT2S0: O_3_ concentration 110 ± 10 ppb and no straw return at the podding stage; BFT2S1: O_3_ concentration 110 ± 10 ppb and straw return at the podding stage; SOC: soil organic carbon concentration; DON: dissolved organic nitrogen; MBC: microbial biomass carbon; MBN: microbial biomass nitrogen; MBP: microbial biomass phosphorus; *P < 0.05; **P < 0.01; ***P < 0.001.

O_3_ stress at a concentration of 80 ± 10 ppb (T1 treatment) significantly increased SOC at the branching stage, while at the podding stage, SOC significantly decreased under the T1 treatment compared with the control (O_3_ concentration 45 ± 10 ppb and no straw return treatment; T0S0; *P* < 0.05) (**Figure 1A**). SOC was significantly increased by straw return under the T1 treatment. According to the results of the GLM, soil DON concentration was not significantly influenced by O_3_ stress (**Figure 1B**; *P* < 0.05). Soil DON concentration was significantly increased by straw return at the branching stage but significantly decreased by straw return at the podding stage. Soil P concentration significantly decreased under the T1 treatment during the whole growth stage compared with the control, and soil P concentration was significantly increased by straw return under the T1 treatment (**Figure 1C**; *P* < 0.05). The O_3_ stress showed insignificantly effects on soil pH (**Figure 1D**; P>0.05). O_3_ stress at a concentration of 110 ± 10 ppb (T2 treatment) significantly increased soil microbial biomass at the branching stage and decreased soil microbial biomass at the podding stage (**Figures 1E–G**; *P* < 0.05). Soil microbial biomass C and soil microbial biomass N were significantly decreased by straw return at the branching stage, while at the podding stage, both microbial biomass C and microbial biomass N were significantly increased by straw return under the T2 treatment (**Figures 1E, F**; *P* < 0.05). Soil microbial biomass P was significantly increased by straw return under the T2 treatment during the whole growth stage (**Figure 1G**; *P* < 0.05).

Overall, O_3_ stress significantly decreased invertase activity and polyphenol oxidase activity at the podding stage (**Figures 1H, I**; *P* < 0.05). Invertase activity and polyphenol oxidase activity were significantly increased by straw return under O_3_ stress during the whole growth stage. Peroxidase activity was significantly higher under the T1 treatment and lower under the T2 treatment at the branching stage, whereas it was significantly lower under the T1 treatment and higher under the T2 treatment at the podding stage compared with the control (**Figure 1J**; *P* < 0.05). Peroxidase activity was significantly decreased by straw return under the T2 treatment during the whole growth stage. Acid phosphomonoesterase activity was significantly higher under the T1 treatment compared with the control during the whole growth stage (**Figure 1L**; *P* < 0.05). Acid phosphomonoesterase activity was significantly decreased by straw return under the T1 treatment and increased by straw return under the T2 treatment during the whole growth stage.

### 3.2 Microbial diversity and microbial community composition

**Figure 2:**
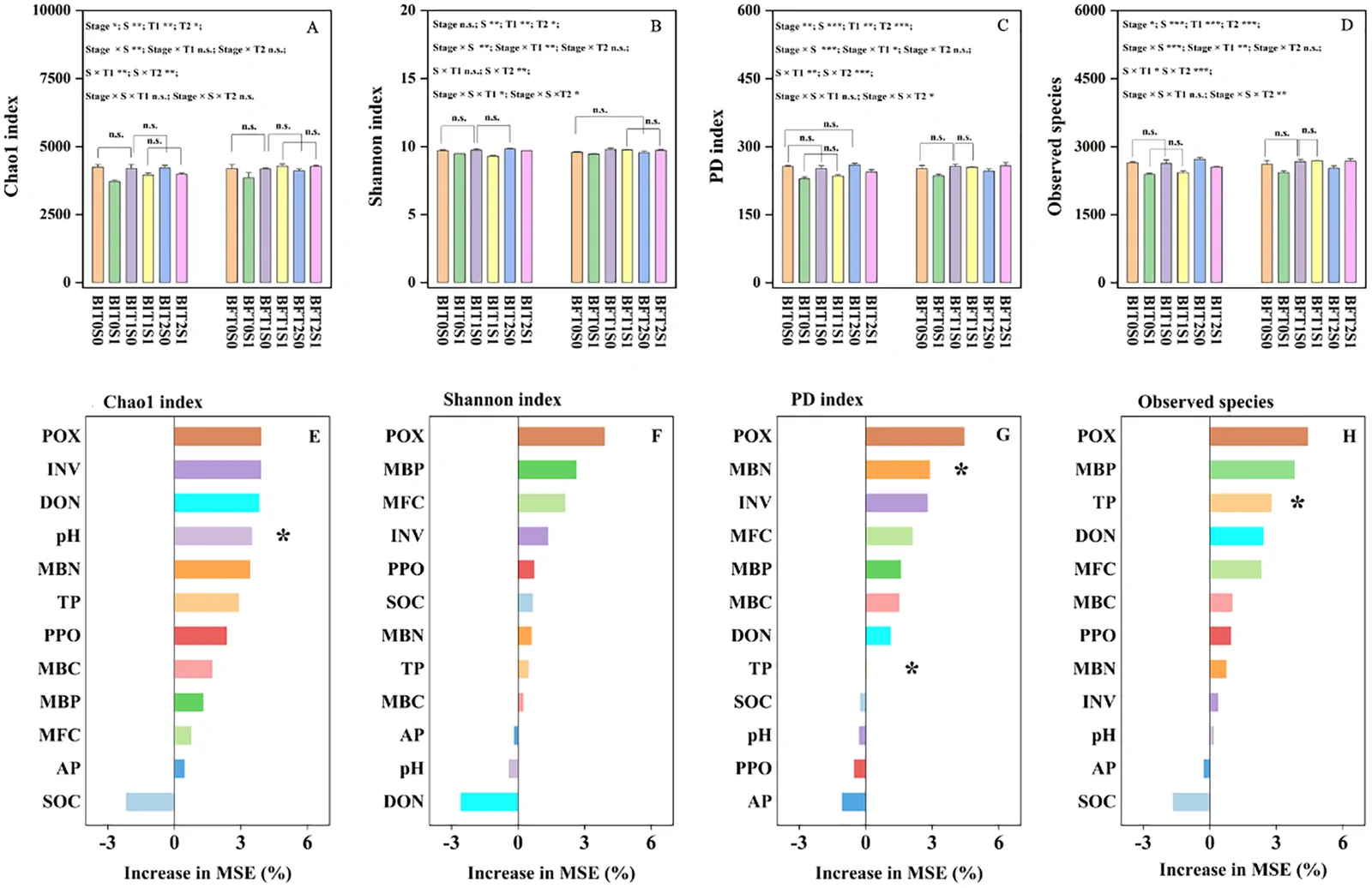
Soil microbial diversity **(A–D)** and the results of Random Forest analysis **(E–H)** under O_3_ stress and straw return at the branching and podding stages. Data are shown as mean ± standard errors (n = 3). For each parameter, results of the general linear model (GLM) are reported, with asterisks showing the significant effect of O_3_ stress (T1: O_3_ concentration 80 ± 10 ppb; T2: O_3_ concentration 110 ± 10 ppb), straw return (S), growth stage (stage), and their interactions. BIT0S0: O_3_ concentration 45 ± 10 ppb and no straw return at the branching stage; BIT0S1: O_3_ concentration 45 ± 10 ppb and straw return at the branching stage; BIT1S0: O_3_ concentration 80 ± 10 ppb and no straw return at the branching stage; BIT1S1: O_3_ concentration 80 ± 10 ppb and straw return at the branching stage; BIT2S0: O_3_ concentration 110 ± 10 ppb and no straw return at the branching stage; BIT2S1: O_3_ concentration 110 ± 10 ppb and straw return at the branching stage; BFT0S0: O_3_ concentration 45 ± 10 ppb and no straw return at the podding stage; BFT0S1: O_3_ concentration 45 ± 10 ppb and straw return at the podding stage; BFT1S0: O_3_ concentration 80 ± 10 ppb and no straw return at the podding stage; BFT1S1: O_3_ concentration 80 ± 10 ppb and straw return at the podding stage; BFT2S0: O_3_ concentration 110 ± 10 ppb and no straw return at the podding stage; BFT2S1: O_3_ concentration 110 ± 10 ppb and straw return at the podding stage; *P < 0.05; **P < 0.01; ***P < 0.001. Random Forest analysis **(E–H)** evaluating the potential correlation among soil physicochemical and biochemical properties and soil microbial diversity. SOC, soil organic carbon concentration; DON, dissolved organic nitrogen; TP, P concentration; MBC, microbial biomass carbon; MBN, microbial biomass nitrogen; MBP, microbial biomass phosphorus; INV, invertase activity; PPO, polyphenol oxidase activity; POX, peroxidase activity; AP, acid phosphomonoesterase activity; *P < 0.05.

Chao1 index, Shannon index, PD index, and observed species were significantly decreased by straw return at the branching stage under O_3_ stress (**Figures 2A–D**; *P* < 0.05). Straw return significantly increased Chao1 index, Shannon index, PD index, and observed species at the podding stage under the T2 treatment. We identified the main controlling factors of soil physicochemical and biochemical properties and enzyme activity affecting microbial diversity by Random Forest (RF) analysis (**Figures 2E–H**). The results showed that soil pH was significantly related to the Chao1 index, and total P concentration had a significant correlation with the PD index and observed species.

**Figure 3:**
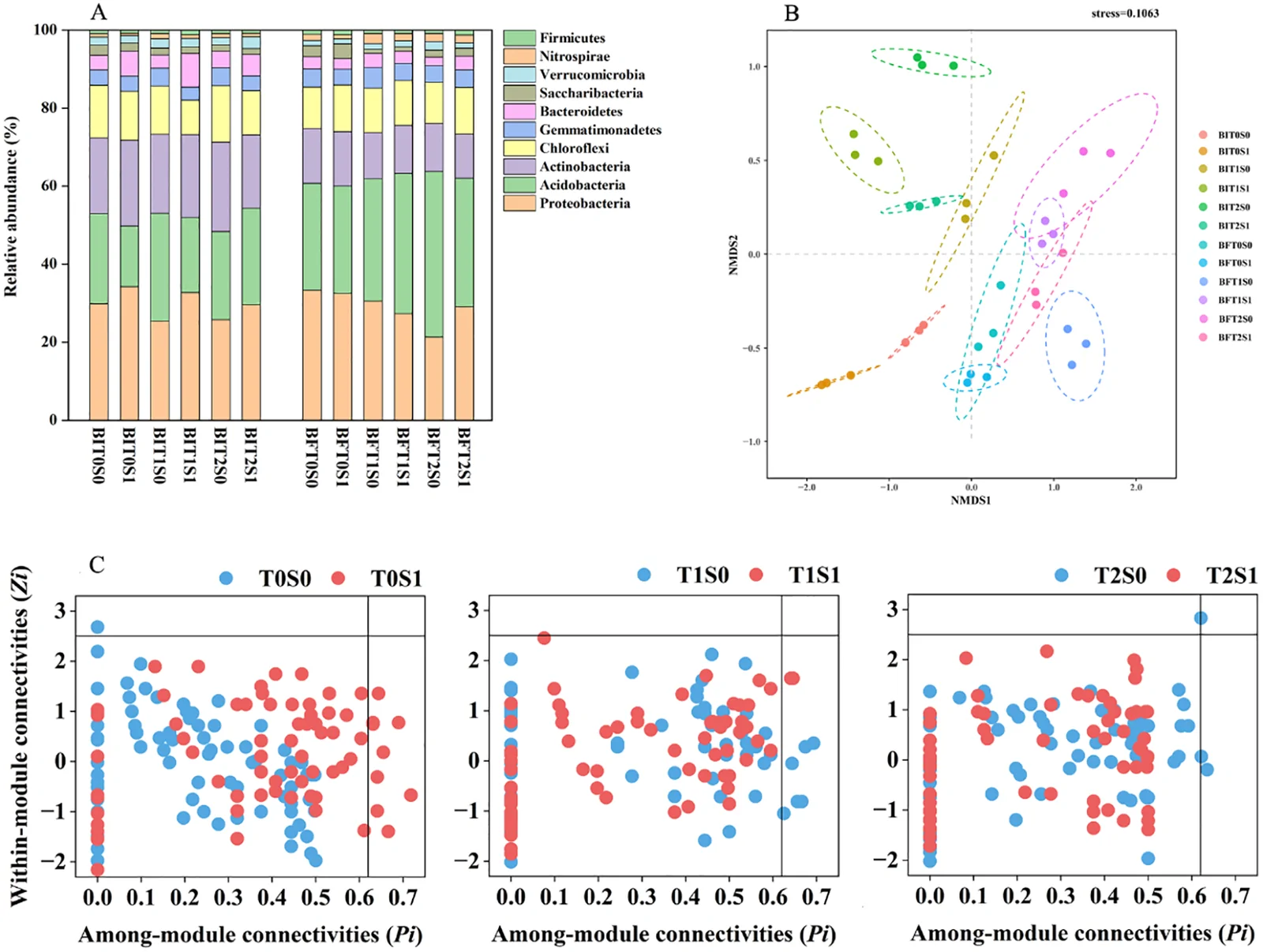
Differences in microbial community composition (phylum level) under O_3_ stress and straw return at the branching and podding stages **(A)**. Non-metric multidimensional scaling (NMDS) analysis comparing differences in the microbial community composition under O_3_ stress and straw return at the branching and podding stages **(B)**. BIT0S0: O_3_ concentration 45 ± 10 ppb and no straw return at the branching stage; BIT0S1: O_3_ concentration 45 ± 10 ppb and straw return at the branching stage; BIT1S0: O_3_ concentration 80 ± 10 ppb and no straw return at the branching stage; BIT1S1: O_3_ concentration 80 ± 10 ppb and straw return at the branching stage; BIT2S0: O_3_ concentration 110 ± 10 ppb and no straw return at the branching stage; BIT2S1: O_3_ concentration 110 ± 10 ppb and straw return at the branching stage; BFT0S0: O_3_ concentration 45 ± 10 ppb and no straw return at the podding stage; BFT0S1: O_3_ concentration 45 ± 10 ppb and straw return at the podding stage; BFT1S0: O_3_ concentration 80 ± 10 ppb and no straw return at the podding stage; BFT1S1: O_3_ concentration 80 ± 10 ppb and straw return at the podding stage; BFT2S0: O_3_ concentration 110 ± 10 ppb and no straw return at the podding stage; BFT2S1: O_3_ concentration 110 ± 10 ppb and straw return at the podding stage. Putative keystone species of soil microbial community (phylum level) under O3 stress and straw return at branching and podding stages **(C)**. T0S0: O_3_ concentration 45 ± 10 ppb and no straw return; T0S1: O_3_ concentration 45 ± 10 ppb and straw return; T1S0: O_3_ concentration 80 ± 10 ppb and no straw return; T1S1: O_3_ concentration 80 ± 10 ppb and straw return; T2S0: O_3_ concentration 110 ± 10 ppb and no straw return; T2S1: O_3_ concentration 110 ± 10 ppb and straw return.

The six most abundant bacteria phyla detected were *Acidobacteria*, *Proteobacteria*, *Actinobacteria*, *Chloroflexi*, *Gemmatimonadetes*, and *Bacteroidetes* (**Figure 3A**). The abundance of *Proteobacteria* decreased under O_3_ stress, and it was significantly increased by straw return under the T2 treatment during the whole growth stage. The abundance of *Acidobacteria* increased under O_3_ stress at a concentration of 80 ± 10 ppb (T1 treatment) during the whole growth stage, and straw return increased the abundance of *Acidobacteria* at the branching stage but decreased its abundance at the podding stage under the T1 treatment. The results of the NMDS analysis indicated significant differences in microbial community structures among the six treatments in the present study (**Figure 3B**). The keystone species in the bacterial network were *Verrucomicrobia*, *Actinobacteria*, *Proteobacteria*, *Acidobacteria*, and *Chloroflexi* acting as connectors in the T0S1 treatment; *Gemmatimonadetes*, *Nitrospirae*, *Proteobacteria*, *Acidobacteria*, *Actinobacteria*, and *Chloroflexi* acting as connectors in the T1S0 treatment; and *Proteobacteria* acting as the connector in the T1S1 treatment (**Figure 3C**).

**Figure 4:**
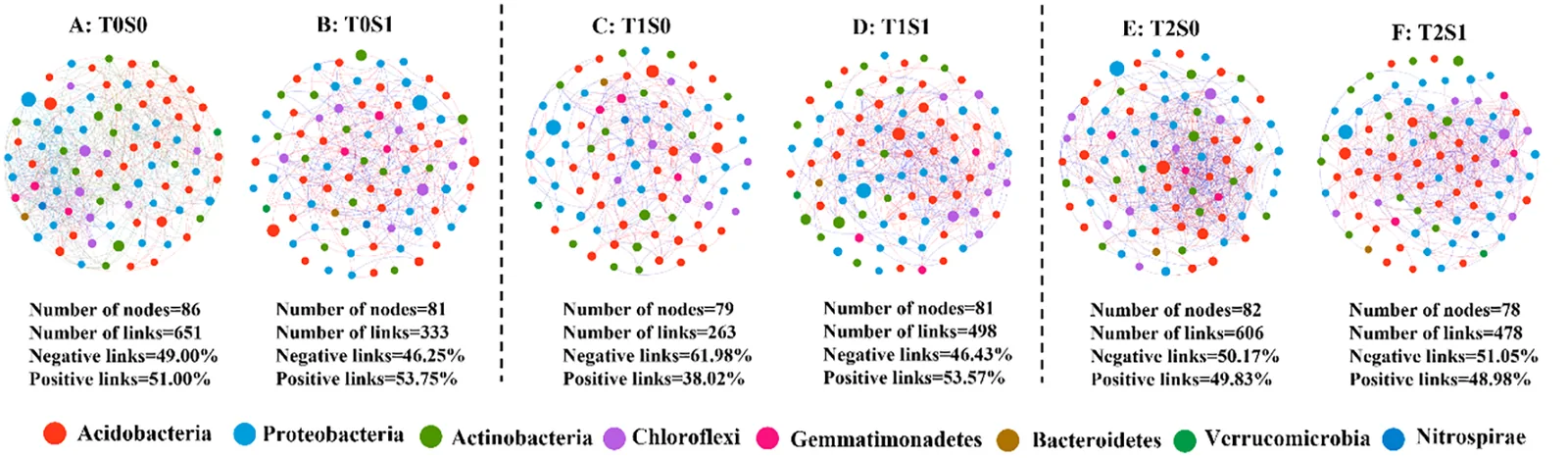
Network of co-occurring bacterial OTUs across the effects of O_3_ stress and straw return. Only Spearman's correlation coefficient (r > 0.7 or r < -0.7 significant at P < 0.01) is shown. Thenodes are colored according to phyla. The number of nodes is the average abundance of the OUT of bacterialphyla. Red lines indicate positive relationships (positive links), and blue lines indicate negative relationships (negative links). TOS0 **(A)**: O_3_ concentration 45±10 ppb and no straw return; TOS1 **(B)**: O_3_ concentration 45±10 ppb and strawreturn; TIS0 **(C)**: O_3_ concentration 80±10 ppb and no straw return; TIS1 **(D)**: O_3_ concentration 80±10ppb and straw return; T2S0 **(E)**: O_3_ concentration 110±10 ppb and no straw return; T2S1 **(F)**: O_3_ concentration 110±10 ppb and straw return.

A co-occurrence network was used to identify closely related bacterial communities. The number of nodes was higher in the T0S0 treatment compared with the T1S0 and T2S0 treatments (**Figures 4A, C, E**). Thus, the number of nodes was decreased by O_3_ stress. In contrast, the number of links was lower in the T0S1 treatment than in the T1S1 treatment (333 vs 498; **Figures 4B, D**). The number of nodes was higher in the T0S1 treatment compared with the T2S1 treatment (78 vs 81; **Figures 4B, F**). Furthermore, the number of average degree (avg K) was decreased by O_3_ stress, and increased by straw return under O_3_ stress (**Table 1**).

**Table 1:** Network topological characteristics of the responses of soil microbial community composition to O_3_ stress and straw return at the branching and podding stages.

Parameter	T0S0	T0S1	T1S0	T1S1	T2S0	T2S1
Average degree (avg K)	15.14	8.222	6.658	12.296	14.78	12.256
Average clustering coefficient (avg CC)	0.482	0.342	0.334	0.528	0.548	0.518
Average path distance (GD)	2.204	2.825	3.013	2.627	2.545	2.563
Geodesic efficiency (E)	0.526	0.428	0.407	0.473	0.497	0.484
Harmonic geodesic distance (HD)	1.9	2.338	2.458	2.116	2.014	2.068
Centralization of degree (CD)	0.155	0.177	0.149	0.278	0.269	0.29
Centralization of stress centrality (CS)	0.217	0.407	0.433	1.244	0.706	0.923
Maximal eigenvector centrality	0.222	0.276	0.272	0.244	0.217	0.255
Centralization of eigenvector centrality (CE)	0.145	0.195	0.193	0.164	0.14	0.175
Density (D)	0.178	0.103	0.085	0.154	0.182	0.159
Transitivity (Trans)	0.514	0.344	0.349	0.551	0.642	0.584
Efficiency	0.832	0.908	0.918	0.857	0.819	0.844

T0S0: O_3_ concentration 45 ± 10 ppb and no straw return; T0S1: O_3_ concentration 45 ± 10 ppb and straw return; T1S0: O_3_ concentration 80 ± 10 ppb and no straw return; T1S1: O_3_ concentration 80 ± 10 ppb and straw return; T2S0: O_3_ concentration 110 ± 10 ppb and no straw return; T2S1: O_3_ concentration 110 ± 10 ppb and straw return.

**Figure 5:**
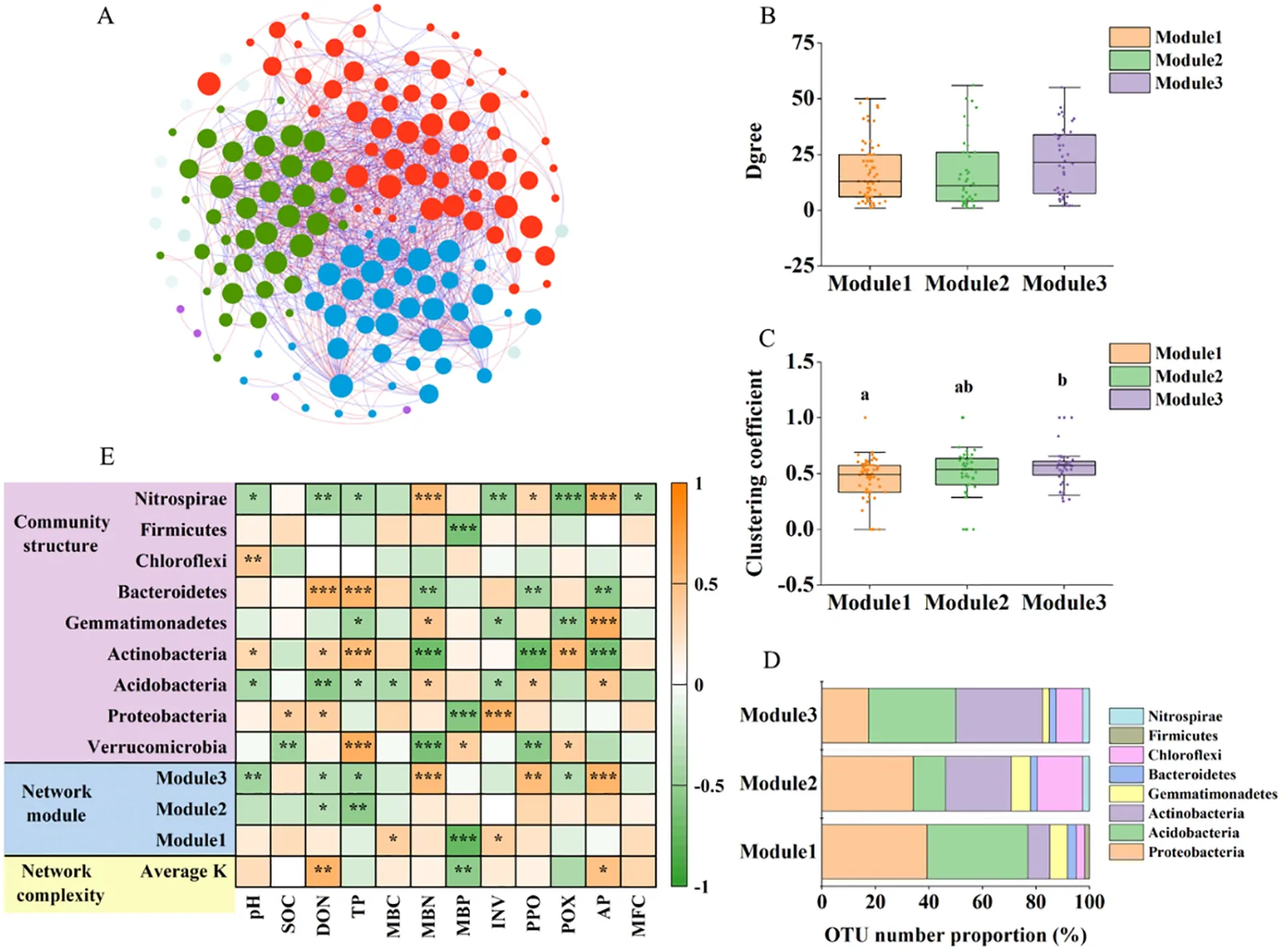
The symbiosis modules are based on a co-occurrence network. The most distinct symbiosis modules of the co-occurrence network (Modules 1, 2, and 3) **(A)**. The difference of degree **(B)** and clustering coefficient **(C)** in Modules 1, 2, and 3. The composition of microorganisms in Modules 1, 2, and 3 **(D)**. Correlation coefficients between the abundance of the nine phyla, Modules 1–3, network complexity, and soil physicochemical and biochemical properties **(E)**. SOC, soil organic carbon concentration; DON, dissolved organic nitrogen; TP, P concentration; MBC, microbial biomass carbon; MBN, microbial biomass nitrogen; MBP, microbial biomass phosphorus; INV, invertase activity; PPO, polyphenol oxidase activity; POX, peroxidase activity; AP, acid phosphomonoesterase activity; *P < 0.05; **P < 0.01; ***P < 0.001.

The co-occurrence network had 147 nodes and 1,290 significant edges (P < 0.05) (**Figure 5A**). In the co-occurrence network, the three symbiotic modules (Modules 1, 2, and 3) showed strong co-occurrence. Module 1 had 61 nodes and 496 significant edges, Module 2 had 41 nodes and 300 significant edges, and Module 3 had 40 nodes and 393 significant edges. We conducted separate network analyses for Modules 1, 2, and 3 and discovered that Module 3 had a significantly higher clustering coefficient than Module 1. In contrast, there were no significant differences in degree among Modules 1, 2, and 3 (**Figures 5B, C**). It indicated that Module 1 was mainly formed by *Proteobacteria* (39.3%) and *Acidobacteria* (37.7%); Module 2 mainly included *Proteobacteria* (34.1%), *Actinobacteria* (24.4%), and *Chloroflexi* (17.1%); and *Acidobacteria* (32.5%) and *Actinobacteria* (32.5%) accounted for 65.0% of the total nodes in Module 3 (**Figure 5D**). Module 1 was significantly related to soil MBC, MBP, and invertase activity (**Figure 5E**). Modules 2 and 3 were significantly correlated with soil DON concentration and soil P concentration. Network complexity had a significant relationship with DON concentration, MBP, and soil acid phosphomonoesterase activity.

### 3.3 Influential factors affecting microbial community and network complexity

**Figure 6:**
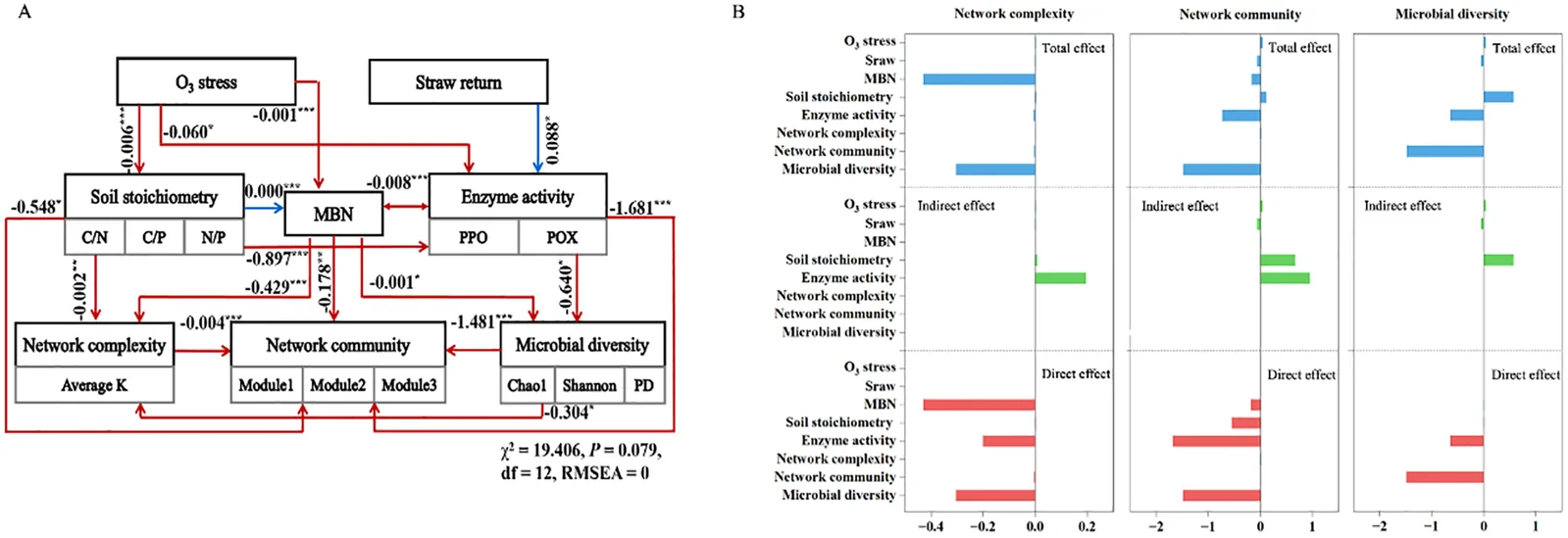
Structural equation model (SEM) illustrating the direct and indirect effects of O_3_ stress and straw return on soil stoichiometry, MBN, soil enzyme activity, network complexity, network community, and microbial diversity **(A)**. Red lines represent significant negative relationships and blue lines represent significant positive relationships. Numbers adjacent to arrows are standardized path coefficients. Significance levels are denoted with *P < 0.05, **P < 0.01, ***P < 0.001. Standardized total effects (direct plus indirect effects), direct effects, and indirect effects calculated by the SEMs **(A)** are displayed in **(B)**, respectively. MBN, microbial biomass nitrogen; PPO, polyphenol oxidase activity; POX, peroxidase activity; Chao1, Chao1 index; Shannon, Shannon index; PD, PD index.

SEM was used to better understand the effects of O_3_ stress and straw return on soil physicochemical and biochemical properties and bacterial community composition and diversity (**Figure 6**). The network community was directly and negatively associated with soil stoichiometry, enzyme activity, and microbial diversity (**Figure 6B**). Overall, bacterial diversity and MBN were the two most important factors related to bacterial network complexity, showing higher standardized total effects than soil stoichiometry, enzyme activity, and network community. Furthermore, soil stoichiometry, enzyme activity, and network community showed significant total effects on bacterial diversity. Meanwhile, O_3_ stress exhibited indirect influences on network community and bacterial diversity via soil stoichiometry, MBN, and soil enzyme activity, whereas straw return showed indirect influences on network community and bacterial diversity via soil enzyme activity.

## 4 Discussion

### 4.1 Effects of O_3_ stress and straw return on soil bacterial biomass and microbial diversity

It has been widely accepted that elevated O_3_ significantly decreases soil bacterial biomass and diversity due to reduced carbon allocation and nutrient availability (Agathokleous et al., 2016, 2020; Chen et al., 2019). In contrast, in the present study, SOC, DON concentration, and microbial biomass C and N were significantly increased by O_3_ stress at a concentration of 110 ± 10 ppb at the branching stage, whereas they were significantly decreased by O_3_ stress at a concentration of 110 ± 10 ppb at the podding stage. Compared with the control (O_3_ concentration of 45 ± 10 ppb), O_3_ stress at a concentration of 80 ± 10 ppb showed no significant effects on SOC and MBN at the branching stage or on DON concentration, MBC, and MBN at the podding stage. Thus, the changes in soil physicochemical properties and bacterial biomass caused by O_3_ stress depended on the O_3_ concentration and the soybean growth stage. Furthermore, soil microbial diversity was generally not significantly affected by O_3_ stress at a concentration of 80 ± 10 ppb, consistent with previous studies (Dunbar et al., 2014; He et al., 2014; Wang et al., 2023). The reason was ascribed to the idea that the soil bacterial diversity was generally resilient despite a range of soil chemical and physical changes associated with plant responses to global atmospheric change (Dunbar et al., 2012; Wang et al., 2023). However, under O_3_ stress at a concentration of 110 ± 10 ppb, the PD index and observed species were significantly decreased. Therefore, microbial diversity was inhibited by the high level of O_3_ stress, while it was not significantly affected by the lower levels of O_3_ stress in the present study.

Straw return has been widely recommended as an environmentally friendly management practice to enhance soil C, N, and P concentrations and hence mitigate the damage caused by environmental change (Zhao et al., 2020). In the present study, straw return improved soil phosphorus concentration and pH and activated soil invertase activity exposed to O_3_ stress overall. Previous studies have indicated that straw return would benefits bacterial community growth and diversity (Guo et al., 2016; Jensen et al., 2021). Similarly, under O_3_ stress, the Chao1 and PD indices, along with the number of observed species, were significantly increased by straw return in the present study. According to the Random Forest results, bacterial α-diversity showed significant positive correlations with soil P concentration, pH, and MBN. For instance, the Chao1 index was significantly correlated with soil pH. The PD index had a significant relationship with soil P concentration and MBN. Observed species had a significant relationship with soil P concentration. Thus, under O_3_ stress, the changes in soil microbial diversity induced by straw return may be associated with soil phosphorus concentration, pH, and MBN, consistent with the findings of Liu et al. (2014).

### 4.2 Effects of O_3_ stress and straw return on the soil bacterial community

The most keystone species were *Proteobacteria*, *Acidobacteria*, *Actinobacteria*, and *Chloroflexi* in the present study. Overall, O_3_ stress inhibited the growth of copiotrophic bacteria (Actinobacteria and Proteobacteria) and promoted the growth of oligotrophic bacteria (*Acidobacteria*) at the podding stage. These findings indicate that O_3_ stress was not conducive to the growth of copiotrophic bacteria but favored the growth of oligotrophic bacteria at the podding stage. This might be due to the negative effects of O_3_ stress on soil carbon allocation and nutrient availability at the podding stage of soybean. In contrast, Wang et al. (2021) found that no significant difference was observed under elevated O_3_ treatment at the phylum level in the soil of poplar saplings. These inconsistent results might be due to differences in species and soil quality (Innes et al., 2004; Tomazelli et al., 2023). Furthermore, with straw return, the abundance of *Proteobacteria* decreased under O_3_ stress at a concentration of 80 ± 10 ppb and increased under O_3_ stress at a concentration of 110 ± 10 ppb at the podding stage. Straw return significantly increased the abundance of *Actinobacteria* and *Acidobacteria* under O_3_ stress at a concentration of 80 ± 10 ppb and decreased their abundance under O_3_ stress at a concentration of 110 ± 10 ppb at the podding stage. Thus, the microbial community composition was significantly altered by O_3_ stress and/or straw return, and these changes depended on the O_3_ concentration and the soybean growth stage. Similarly, previous studies have demonstrated that straw incorporation significantly altered soil microbial community structures, and the change of soil microbial responses to straw incorporation depend on agricultural cultivation, soil quality, species, and growth stage (Xia et al., 2019; Cong et al., 2020; Li et al., 2022; Xu et al., 2023).

In addition, *Acidobacteria* are associated with many gene sequences involved in N fixation and C cycling (Lennon and Jones, 2011; Jimenez et al., 2012) and are strongly regulated by extracellular enzymes and soil pH (Costa et al., 2020; Jones et al., 2009). In the present study, the abundance of *Proteobacteria* and *Acidobacteria* had a significant relationship with soil invertase activity and DON concentration. Meanwhile, *Actinobacteria* are an oligotrophic taxon that preferentially utilizes low-quality organic C and may play a role in the degradation of aromatic compounds (Fierer et al., 2007; Wallenstein et al., 2007). Soil DON concentration, pH, MBN, and polyphenol oxidase activity were significantly related to the abundance of *Acidobacteria* and *Actinobacteria* in the present study. *Chloroflexi*, which often participate in the straw decomposition progress, including the degradation of starch and cellulose (Castro et al., 2019), are known to have a close relationship with total phosphorus concentration (Padhy et al., 2021). However, the results of the present study showed that the abundance of *Chloroflexi was* significantly related to soil pH but not to total phosphorus concentration. Therefore, the changes in microbial composition probably resulted from differences in soil pH and chemical and biochemical properties, which were likely attributable to the input of straw and soybean root exudates (Ye et al., 2023). However, root exudates were not investigated in this study. Thus, further studies are needed to provide insights into the regulatory mechanisms of root exudates on microbial ecological processes under O_3_ stress and/or straw return.

### 4.3 Effects of elevated O_3_ and straw return on co-occurrence of microbial community

Co-occurrence networks can be used to investigate correlations among microbial groups under different environmental conditions (Banerjee et al., 2018; Bai et al., 2022; Wang et al., 2023). Network links reflect the significant and strong correlations among species, and fewer links represent reduced function and simplified species connections (Newman, 2006). The degrees of complexity and centralization indicate the ability of the species-species network to avoid stringing collapses when it undergoes environmental stresses (Deng et al., 2012; Jalili and Perc, 2017). In the present study, straw return slightly increased the total links exposed to the O_3_ stress at branching stage, while straw return significantly decreased the total links exposed to O_3_ stress a concentration of 110 ± 10 ppb at the podding stage. These results indicate that changes in the structural complexity of the soil bacterial community also depended on the O_3_ concentration and the soybean growth stage.

Consistent with the results of the co-occurrence network, there was a negative effect of O_3_ stress on the structural complexity of the soil bacterial community, similar to the findings of Wang et al. (2023). Additionally, O_3_ stress significantly decreased soil invertase activity and polyphenol oxidase activity at the podding stage in the present study. These reductions in soil microbial function potentially altered soil C quantity and utilization efficiency, and thus induced the negative impacts of O_3_ stress on the network complexity of the soil bacterial community (He et al., 2014; Agathokleous et al., 2020). Furthermore, straw return positively affected the structural complexity of the soil bacterial community exposed to O_3_ stress at a concentration of 80 ± 10 ppb. Straw incorporation also increased the proportion of positive associations in the networks. Thus, straw return strengthened species associations among microbial members exposed to O_3_ stress at a concentration of 80 ± 10 ppb. However, straw return negatively affected the structural complexity of the soil bacterial community under O_3_ stress at a concentration of 110 ± 10 ppb. Therefore, straw return alleviated the negative effects of O_3_ stress on the structural complexity of the soil bacterial community only under low levels of O_3_ stress, which differs from our Hypothesis 2.

Structural equation modeling was used to examine the direct and indirect effects of O_3_ stress and straw return on soil microbial diversity and community in the present study. Microbial diversity (Chao1, Shannon, and PD indices) and MBN were the key factors affecting the microbial network community (Modules 1, 2, and 3) and complexity (average K), as they showed higher total standard effects than soil stoichiometry (C/N, C/P, and N/P ratios). In addition, indirect effects of soil enzyme activity (polyphenol oxidase activity and peroxidase activity) on microbial network community and complexity were also found in the present study. These results demonstrated that O_3_ stress affected the microbial network community and complexity indirectly via MBN and soil enzyme activity. Furthermore, straw return had the lowest total standard effect on microbial network community and complexity among all factors, indicating that the mitigation effect of straw return on O_3_ stress-induced damage was limited, consistent with our previous study (Mao et al., 2022). According to the SEM results, the slight mitigating effects of straw return on soil microbial network community and complexity were indirectly associated with soil enzyme activity and MBN, contrary to our third hypothesis. Taken together, the combined effects of O_3_ stress and straw return on microbial network community and complexity were not mainly associated with soil physicochemical properties but more generally with microbial function.

## 5 Conclusion

This study provides insight into the influence of O_3_ stress and/or straw return on soil microbial network community and complexity at the branching and podding stages. First, under O_3_ stress, the modifying effects of straw return on soil microbial diversity were associated with the soil P concentration, pH, and MBN. In addition, the four most abundant phyla were *Proteobacteria*, *Actinobacteria*, *Acidobacteria*, and *Chloroflexi*. O_3_ stress negatively affected the structural complexity of the soil bacterial community, and straw return strengthened species associations among microbial members only under low levels of O_3_ stress (80 ± 10 ppb). Finally, O_3_ stress showed indirect negative effects on network community and complexity via MBN and soil enzyme activity. Straw return exerted slight mitigating effects on network community and complexity, which might be indirectly associated with soil enzyme activity and MBN. Collectively, the slight mitigating effects of straw return on the reduced microbial topology were not mainly associated with soil physicochemical properties but more generally with microbial function.

## Data Availability

The datasets presented in this study can be found in online repositories. The names of the repository/repositories and accession number(s) can be found below: https://www.ncbi.nlm.nih.gov/, PRJNA704249.
